# 64-Slice spiral CT perfusion combined with vascular imaging of acute ischemic stroke for assessment of infarct core and penumbra

**DOI:** 10.3892/etm.2013.1107

**Published:** 2013-05-09

**Authors:** DANG-ZHEN BAO, HUAN-YING BAO, LI-ZHAI YAO, YUN-GAO PAN, XIN-RUI ZHU, XIAO-SONG YANG, HE WANG, YI-NING HUANG

**Affiliations:** 1 Department of Neurology, Nanle Rehabilitation Hospital, Puyang, Henan 457400;; 2 Department of Neurology, Nanle Central Hospital, Puyang, Henan 457400;; 3 Department of Neurology and Medical Imaging, Peking University First Hospital, Beijing 100034, P.R. China

**Keywords:** computed tomography, perfusion, penumbra, stroke

## Abstract

The aim of this study was to determine the value of computed tomography perfusion (CTP) parameters, including cerebral blood flow (CBF), cerebral blood volume (CBV), mean transit time (MTT) and time-to-peak (TP), in a clinical study of patients with stroke. Additionally, we determined which parameter or combination of parameters are reliable in detecting the presence of an infarct and penumbra. CTP was performed within 24 h of the onset of symptoms in 20 patients with possible stroke. Magnetic resonance imaging (MRI) was performed 3-7 days later and the threshold of the CTP was adjusted according to the results to provide CT images that correlated with the MRI; the MRI results were taken as the gold standard. CBV, CBF and TP contrast agent enhancement were calculated using the CT results. The CTP results were compared with the MRI findings. All CTP parameters were reliable in detecting the penumbra (P<0.001). In these parameters, changes of MTT were the most useful. CTP revealed various changes in CBF, CBV, MTT and TP in ischemic areas. CTP parameters were also reliable in detecting the infarct core (P<0.001). We determined that when detecting the penumbra, all CTP parameters are reliable, and when detecting cerebral ischemia, a combination of parameters should be used.

## Introduction

Normal physiological function and a variety of pathological changes to human brain tissue are closely associated with changes in cerebral blood flow (CBF); therefore, access to hemodynamic information on human tissues has attracted the attention of imaging researchers (1–5). The theoretical basis is derived from the use of data-processing technology to generate cerebral perfusion images with radionuclides. Radioactive tracers are administered as a bolus injection into a vein, from where they enter the left heart and travel to the back of the head. A time-density curve (TDC) is obtained by dynamic scanning when the tracer passes through the organ for the first time. Brain computed tomography perfusion imaging (CTPI) is also performed for continuous dynamic scanning of the layer in the region of interest (ROI) within a certain time after the intravenous injection of the contrast agent. When the contrast agent reaches the brain tissue, its density gradually increases, peaks and then gradually decreases within a certain time, until the contrast is restored to the density level in the brain tissue prior to injection. The TDC for the contrast agent is obtained by measuring density values in the brain tissue at various times during the CT scan, as well as hemodynamic parameters, including regional CBF (rCBF), regional cerebral blood volume (rCBV), mean transit time (MTT) and time-to-peak (TP). The perfusion images for CBF, CBV, MTT and TP of brain tissue are obtained following pseudo-color processing. The brain CTPI reflects the changes in physiological functions of brain tissue; therefere, it is a functional imaging technique. Pseudo-color images of multiple parameters are obtained within minutes of processing the images with perfusion software, in a similar manner to magnetic resonance perfusion imaging (MRPI). It is a multi-parameter imaging method that is the preferred examination method for ultra-early and early stroke patients.

The central premise of acute stroke thrombolysis is the recovery of the ischemic penumbra (6,7). Although repeated analysis of experimental data by the International Stroke and Neurological Disorder Association has identified no evidence for the efficacy of recombinant tissue plasminogen activator (R-TPA) in the first 3 h (8,9), the data also indicated that thrombolytic therapy beyond 3 h is safe (10). To increase the accuracy of the identification of the ischemic penumbra, extension of the thrombolytic time window is necessary. This has become an urgent requirement for acute stroke imaging. Brain CTP is an effective and convenient method for evaluating acute stroke.

A dynamic scan of several different levels of the brain is obtained through the injection of an iodine-containing contrast agent to perfuse the brain tissue. Based on CT data of dynamic brain perfusion, a number of early studies proposed standards for determining the necrosis or penumbra of the brain tissue. A number of studies considered the absolute value of the CBF (1,2), while others were based on the cerebrovascular autoregulation theory, proposing standards based on a combination of CBF and CBV values (1–5,11,12) to determine the presence of a cerebral infarction core and ischemic penumbra.

The determination of the ischemic penumbra from CTP data has been assessed in a number of studies using various methods. These are often small scale studies using 2-slice, 4-slice or <16-slice CTP machines (13). In the current study, 64-slice spiral CT was selected for the examination of patients with clinically suspected acute ischemic stroke, with the purpose of implementing a systematic assessment of all CTP parameters (CBF, CBV, MTT and TP) for acute stroke patients and also to ensure that these parameters accurately detect cerebral ischemia and the ischemic penumbra.

The current study is a small scale, independent prospective study on the brain perfusion of ischemic stroke, with the purpose of exploring judgment standards for the core area and penumbra of cerebral infarction. In particular, the aim is to explore the judgment criteria under lower temporal resolution (low sampling rate), to facilitate the detection of the penumbra during whole brain perfusion and to promote its application in primary health care sites.

The main purpose of the thrombolytic treatment of stroke patients is to save brain tissue in the ischemic penumbra and this has been demonstrated to be effective and feasible. Studies have demonstrated that thrombolytic therapy performed on patients >3 h after the onset of ischemic stroke is safe and reliable (14,15). Therefore, an accurate determination of the existence and the extent of the penumbra is the basis for the treatment of ischemic patients at various time windows. The blood flow in normal human brain gray matter is ∼50–60 ml/100 g/min (6). In the early stages of brain tissue ischemia, a brain tissue hypoperfusion area exists between the infarction core and normal brain tissue. Although this area of brain tissue demonstrates physiological dysfunction, with no significant cell potassium outflow and energy exhaustion, when the normal blood supply is restored, its electrophysiological function is capable of being restored.

CTP volumetric imaging shows a reversible area surrounding the irreversibly infarcted area. The blood flow in these surrounding areas is between the thresholds for electrical activity failure and membrane failure and is known as the penumbra. The ischemic penumbra is dynamic; it returns to a normal state or may progress into infarction. However, the restoration of the electrophysiological activity of brain tissue has a certain time limit; 3–4 h after the onset of cerebral infarction, the vast majority of the ischemic penumbra develops into an irreversibly infarcted area. Therefore, the focus of studies in previous years has been to save the living brain tissue in the ischemic penumbra, which is the key to reducing or avoiding disability. Koenig *et al* (16) performed brain CT perfusion in patients with acute cerebral ischemia within 6 h of symptom onset and the sensitivity of penumbra detection was 90%, with a specificity of 100%.

The CBF ratio in the cerebral ischemic lesion center may be used to distinguish between reversible and irreversible ischemic areas. A disputed study determined a CBF ratio of 0.2 as the minimum threshold for the survival of ischemic brain tissue and stated that when the CBF ratio is <0.2, the brain tissue has been necrotized, and when the CBF ratio is 0.2–0.35, the ischemic penumbra should be considered (16) and the effect of thrombolytic therapy is clear. Decreased CBF, normal or mildly elevated CBV, abnormal MTT and normal or delayed TP are regarded as characteristics of an abnormal perfusion area (11). MTT extension and a significant decrease of CBF in the cerebral ischemia area indicate irreversible damage; extension of the MTT and maintenance or increase of the CBV also indicate irreversible damage (17). CTP shows the abnormal perfusion areas as early as 30 min after the onset of symptoms (11).

## Patients and methods

### Clinical data

A total of 22 patients with neurological deficit symptoms and with intracerebral hemorrhage ruled out using a CT scan, who were admitted to the Emergency Department of Neurology of Peking University First Hospital (Beijing, China) from August 2005 to June 2006, were retrospectively studied. This study was conducted in accordance with the Declaration of Helsinki and with approval from the Ethics Committee of Peking University First Hospital. Written informed consent was obtained from all participants. Inclusion criteria for the patients were as follows: within 24 h of stroke onset; no previous history of stroke; intracranial hemorrhage determined using a CT scan; and no contraindications of imaging using iodinated contrast agent and contraindications of MRI examination. Of the 22 patients, 13 were male and 9 were female, aged 42–73 years, with a mean age of 56.7 years. The symptoms of the patients manifested as transient, persistent or recurrent ischemic attacks. Of the 22 cases, 8 patients presented episodic limb weakness, 6 patients had dysphonia, 4 patients presented tongue deflection, 2 patients presented dysphonia and unresponsiveness and 2 patients presented aphasia combined with unilateral hemiparesis. All patients received a plain CT scan and the results were confirmed by CTPI and CT angiography (CTA) at 1–24 h after the onset of the disease. All the selected cases underwent MRI examination within 3–10 days of the onset of the disease. This identified that the infarct areas were larger than the range observed during perfusion.

### Initial CT examination

The initial CT examination included CT brain scans, head and neck vascular CTA and 4-slice brain CTP examination (sampling rate of one slice per second). After reprocessing the data, a variety of brain perfusion maps with a sampling rate of one slice per second and with a sampling rate interval of 4 slices per second were obtained. A plain brain scan using 64-slice spiral CT was performed immediately after admission and CTPI and CTA examinations were conducted once cerebral hemorrhage had been excluded. The slice thickness of perfusion imaging was set to 2.5–10 mm (10 or 5 mm for the brain hemisphere and 5 or 2.5 mm for the brainstem or cerebellum). The range of angiography was set from the upper edge of the aortic arch to the calvaria.

### CTPI examination

Patients were asked to avoid head movements and to breathe quietly. The patient’s heads were fixed with straps and a CT plain scan, CTP and CTA were completed within 5–10 min. A 40 ml bolus injection of nonionic contrast agent (370 mg/ml; Ultravist, Bayer Vital GmbH, Berlin, Germany) was administered into the ulnar vein with a Medrad-stellant high-pressure syringe (binoculars) at a flow rate of 4 ml/sec. A synchronous dynamic axial CT scan was performed at the same time as the injection of the contrast agent. The scan parameters were as follows: 120 kV; 400 mAsec; interval, 1 sec; scan time, 50 sec and coverage, 10 mm × 8 slices. A total of 200 images of slices in the ROI were obtained. The CBF of brain tissue which was included in the scan range of 10 mm × 8 slices was calculated. By which the brain blood supply was evaluated.

The slices in which lesions were identified with clinical positioning and CT scanning were selected for assessment and calculation. The processing software in the workstation (Extended Brilliance™ Workspace workstation, Philips, Amsterdam, The Netherlands) was employed for data processing to obtain the TDC of the contrast agent passing through the brain tissue and to calculate the CT parameter values in the selected lesions and corresponding contralateral area. The midline was used as a mirror plane, to symmetrically measure the MTT (sec) of the contrast agent passing through the lesion and the corresponding contralateral area. The rCBF (ml/min/100 g), rCBV (ml/100 g) and TP (sec) of the contrast agent, as well as other hemodynamic parameter values were quantitatively analyzed. On the pseudo-color images obtained from post-processing, the thresholds were adjusted and determined and then the blood vessels that may interfere with the results were removed to avoid skull pseudo-shadow. The CBV, CBF, TP and MTT in the abnormal area marked with red and green colors were displayed and the parameter values in the lesion core and the surrounding area were simultaneously measured, analyzed and compared.

### Follow-up MR examination

Follow-up MR examination included fluid-attenuated inversion recovery (FLAIR), diffusion-weighted imaging (DWI) axial sequence and 3D time-of-flight (TOF) magnetic resonance angiography (MRA). The infarct size was determined according to the final outcome of the MR examination. Finally, indicators for the cerebral perfusion parameters of the cerebral infarction core area and the penumbra at varying sampling rates were proposed.

### Image processing

**Figure 1.**
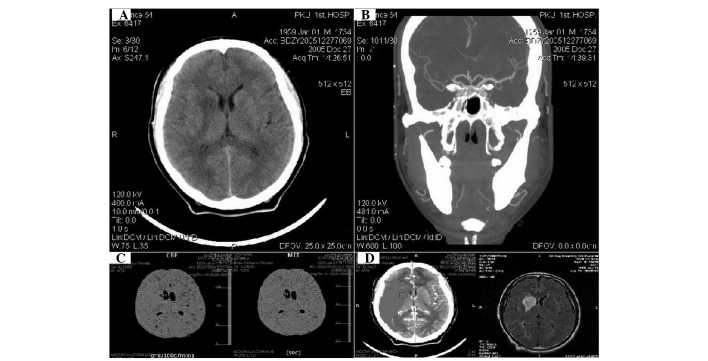
Case 1, male, 46 years old, with mixed aphasia combined with left limb weakness for 5 h. The patient was admitted to hospital and presented no changes in consciousness. (A) A plain computed tomography (CT) scan revealed that cerebral infarction occurred in the right basal ganglia, corona radiata and semiovale center, right temporal lobe and parietal lobe. (B) CT angiography (CTA) revealed that the first portion of the right middle cerebral artery was in severe stenosis. (C) Cerebral blood flow (CBF) in the area of the right brain lesion was significantly reduced; the CBF ratio was <0.2, indicating irreversible ischemia. The CBF ratio around the lesion core was >0.2, indicating the ischemic penumbra. The mean transit time (MTT) of the right brain lesions was extended. (D) An infarct size of infarct area was identified by magnetic resonance (MR)-weighted imaging at <7 days.

**Figure 2.**
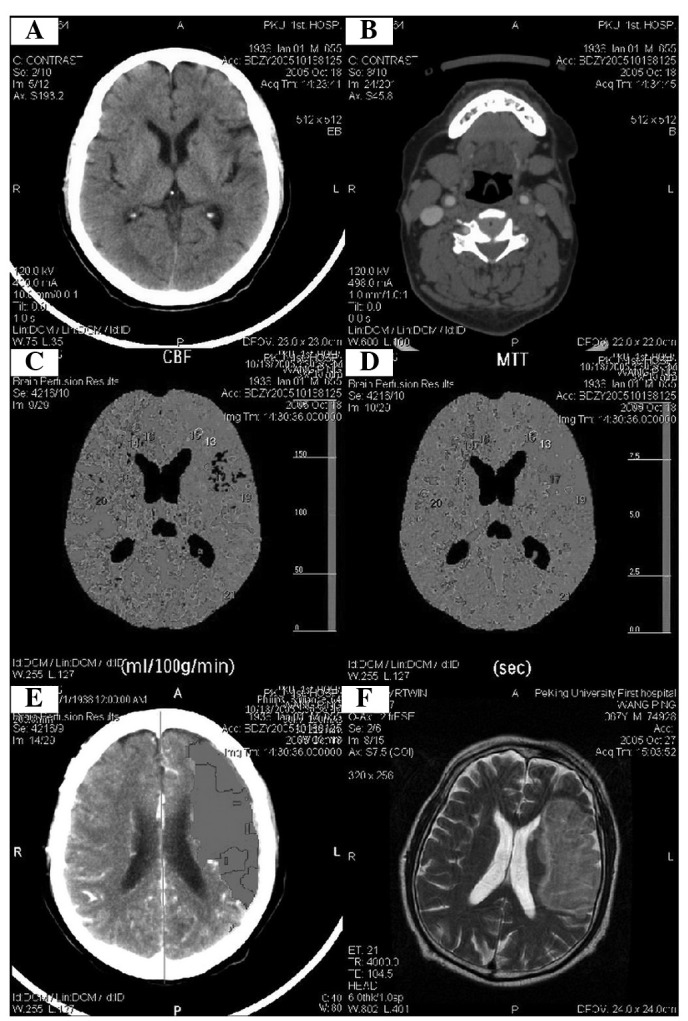
Case 2, male, 67 years old. Language was not fluent and the mouth/nose was skewed when admitted into hospital. The patient presented mixed aphasia with a consciousness trance-like state for 3 h and the right pathology was weak positive. The plain computed tomography (CT) scan revealed that a lacunar infarct occurred in the left frontal hemisphere, head of the left caudate nucleus and the left radiation crown area. However, CT perfusion identified large areas of ischemia in the left hemisphere and a small infarction area in the middle hemisphere. Two days later, limb function gradually accelerated, until complete paralysis and aphasia occurred. (A) New cerebral infarction in the head of the caudate nucleus. (B) Bilateral carotid atherosclerotic stenosis on the left (50%) and right (30%). (C and D) The cerebral blood flow (CBF) ratio around the lesion core of the left hemisphere was <0.2 indicating irreversible ischemic infarction and the area surrounding the core was considered the ischemic penumbra. (E and F) The infarct area was observed after <3 days using magnetic resonance imaging (MRI).

After processing with the Extended Brilliance™ Workspace workstation, the images were jointly interpreted by two radiologists and neurologists with five years’ relevant work experience. In this study, the CTP threshold was adjusted according to the nuclear magnetic resonance (NMR) image, to obtain abnormal perfusion images that were similar to the NMR images and to measure and obtain the parameter values in the area presenting abnormal perfusion. The values of MTT, CBF, CBV, TP and other hemodynamic parameters in the selected lesion and corresponding contralateral areas were recorded. The differences in the values of the hemodynamic parameters in the lesion core and the surrounding area were simultaneously measured (Figs. 1 and 2).

### Statistical analysis

All data were processed with SPSS 11.0 software (SPSS Inc., Chicago, IL, USA). The absolute value of the parameters in the perfusion area and the ratio of the lesion area and contralateral area were calculated. The parameters of the affected side and the control area were assessed with Wilcoxon signed ranks test. The values of the ratio of the lesion area and contralateral area −1 were assessed with an independent sample t-test. P<0.05 was considered to indicate a statistically significant result.

## Results

A significant difference was observed between the absolute values of the parameters in the ischemic penumbra and the corresponding healthy side area, using Wilcoxon signed ranks test (P<0.001; Table I), demonstrating that the absolute values of each parameter reflect lesion change. Among the values of the ratio of the lesion area and contralateral area −1, assessed with the independent sample t-test, the difference between the CBV ratio and the TP ratio was not determined to be significant (P>0.05). The differences between the other parameters were significant (P<0.05; Table II).

Four of the parameters were normally distributed. By comparing the arithmetic mean of the four parameters, we identified that the value of the MTT ratio of the affected side and healthy side −1 (Table III) was the maximum value; therefore, the change in MTT was the maximum change. In the Wilcoxon signed ranks test, a significant difference was observed between the parameters in the infarct lesions and in the healthy side (P<0.001; Table IV). This demonstrated that the absolute value of each parameter is reflective of lesion change.

By calculating the values obtained from the ratio of parameters in the infarct area to the contralateral area −1, we identified that the internal differences of the parameters were large; however, they were not normally distributed (Table V). In these parameters, the direction change of certain parameters does not match with that of the majority of parameters, which may be related to the greater difference of change in early infarct blood flow dynamics. The results indicate that the four parameters should be jointly used when defining and distinguishing infarct lesions.

Our results (Tables IV and V) indicate that the currently available data do not distinguish the pros and cons of the differences in the four parameters. The differences may be due to: i) the screening criteria of the study subjects; ii) the onset time differences in the study subjects; iii) selection of a reasonable MRI and CTP examination time; iv) generation of large differences between the values for different lesion sizes; v) differences in the age and gender of the patients; and vi) differences in the recovery or progress of lesions in the acute phase.

## Discussion

CTP has been reported to be a simple and effective imaging technology for evaluating the scope and extent of the ischemic penumbra in patients with acute stroke in the emergency room (18). CTPI has a diagnostic performance comparable to those of MRI [(DWI and perfusion-weighted imaging (PWI)] and positron emission tomography (PET)-CT; however, a CTP scan is more convenient and efficient to perform in the majority of medical institutions (17–21). Assessment of the penumbra and infarct core relies on a multi-layered dynamic CTP, including the acquisition technology of sequential CT data. This technology is derived from the bolus injection method for intravenous injection of an iodine-containing contrast agent. The 64-slice spiral CTP examination is an effective and easy method for evaluating acute stroke. By injection of an iodine-containing contrast agent, a dynamic scan of the same area in several slices of the brain may be performed to obtain perfusion data of the brain tissue.

Based on CT data of dynamic brain perfusion, a number of studies have proposed standards for determining necrosis or the ischemic penumbra in the brain tissue. A number of studies considered the absolute value of the CBF (1,2), while other studies were based on the cerebrovascular autoregulation theory (1–5,11,12). The latter proposed standards for data, based on combinations of CBF with CBV, to determine the presence of the cerebral infarction core and ischemic penumbra. However, the limited number of studies have certain shortcomings. Firstly, the majority of the studies included a small number of cases (12–22 cases). Secondly, the standard of judgment was set relatively optionally. A study with a relatively large number of cases (130 cases), in which the relative and absolute values of the CBF, CBV, MTT and TP in the images of 25 cases were evaluated by Wintermark and Bogousslavsky (17), with the use of ROC curve analysis, proposed the use of the relative MTT and absolute CBV values as the standard for identifying the cerebral infarction core area and the penumbra.

Results obtained in other studies are quite different from these standards, and reliable reports and standards for corresponding data are lacking; thus, it is difficult to establish a corresponding standard using a certain set of data in the literature. In addition, in one study (22), the method used had a sampling rate of one slice per second (high resolution) and it requires a high radiation dose. Additionally, the primary hospital equipment is difficult to support and is not suitable for a wide range of whole brain perfusion. By reducing the sampling rate of CTP (low resolution), we are able to successfully obtain the parameters of cerebral perfusion. However, normal brain perfusion parameters acquired at low sampling rates have different results in different studies. The current study identified that when time resolution is reduced from one slice per second to an interval of 4 sec per slice, the values of the perfusion parameter CBV decrease, while the TP value is extended. Currently, there is no standard in which a time resolution of 4 sec is used to determine the existence of the penumbra within the brain tissue.

We conducted a small scale study of acute stroke patients using a 64-slice CTP technique and all CTP parameters (CBF, CBV, MTT and TP) were systematically assessed. MRI was used for the final evaluation of the size of the stroke-affected area. The ultimate goal was to determine the penumbra parameters and the composite value of all parameters to produce more accurate predictors of infarction and penumbra.

In this study, CTP examination was conducted within 24 h; however, MR examination was performed after 3–7 days, so there was a time interval between the two results. The difference between the lesion sizes identified in MR examination and the lesion sizes in CTP were analyzed. In addition, based on the results of statistical analysis, the CBV values in the infarction area were not fully in line with theoretical values, which may be a result of the varying amount of local blood supply in the infarction area at the time of examination.

The purpose of this study was to investigate the credibility of the various parameters in identifying the penumbra and infarction area using CTP, rather than to study the consistency of the results of CTP with MR results and the absolute accuracy of MR. In addition, NMR was considered the gold standard; however, it is not the only option, since a second plain CT following perfusion may also be used as the gold standard to confirm the progress and recovery condition shown in the penumbra and infarct.

The combination of 64-slice CTPI and CTA may be used to respectively evaluate cerebral perfusion in 8–32 slices and 80 mm range up and down and may be conducted immediately after the onset of clinical symptoms of ischemic lesions. The CBF of brain tissue which was included in the scan range of 10 mm × 8 slices was calculated. By which the brain blood supply was evaluated. Dynamic CTP of the same area of multiple slices is achieved, with fast scanning and a large scanning range. The automatic mAsec technique reduces the amount of rays related to repeated scans and the use of the respiratory gating technique reduces motion artifacts during detection. We consider that the focal points in the development of the technology to improve the quality of multi-slice brain CT imaging should include: improvement of the physical temporal resolution by increasing the rack speed; improvement of the spatial resolution through the development of thinner detectors and more advanced reconstruction algorithms; improvement of the image quality through research and development of a detector with more slices (for example, 256 slices) or even a flat panel detector; expansion of the adaptive population of 64-slice CT brain imaging through the development of more advanced respiratory gating technology and to better apply the CT imaging technique adopted in very small lesions (including the cerebellum and brainstem). With the continuous development and progress in multi-slice CT technology, this technique is likely to be more widely used in the diagnosis of ischemic stroke.

## Figures and Tables

**Table I.** Wilcoxon signed ranks test between the absolute values of parameters in the ischemic penumbra and corresponding healthy side area.

Parameters	N	Mean rank	Sum of ranks	P-value
CBV2-CBV1				
Negative ranks	77^a^	72.18	5558.00	<0.05
Positive ranks	57^b^	61.18	3487.00	
Ties	1^c^			
Total	135			
CBF2-CBF1				
Negative ranks	12^d^	29.33	352.00	
Positive ranks	123^e^	71.77	8828.00	
Ties	0^f^			
Total	135			
MTT2-MTT1				
Negative ranks	130^g^	68.10	8852.50	
Positive ranks	5^h^	65.50	327.50	
Ties	0^i^			
Total	135			
TP2-TP1				
Negative ranks	130^j^	69.11	8984.00	
Positive ranks	5^k^	39.20	196.00	
Ties	0^l^			
Total	135			

CBV1, CBF1, MTT1 and TP1: ischemic penumbra perfusion parameters; CBV2, CBF2, MTT2 and TP2 healthy side area parameters.

a CBV2 < CBV1;

b CBV2 > CBV1;

c CBV2 = CBV1;

d CBF2 < CBF1;

e CBF2 > CBF1;

f CBF2 = CBF1;

g MTT2 < MTT1;

h MTT2 > MTT1;

i MTT2 = MTT1;

j TP2 < TP1;

k TP2 > TP1;

l TP2 = TP1. CBV, cerebral blood volume; CBF, cerebral blood flow; MTT, mean transit time; TP, time-to-peak.

**Table II.** Independent sample t-test of the ratios of the parameters in the lesions area and the corresponding healthy side area.

Ratio 1	Ratio 2	Mean difference	Standard error	P-value	95% Confidence interval	
					Lower bound	Upper bound
1	2	−0.7788^a^	0.12466	0.000	−1.0237	−0.5339
	3	−1.0264^a^	0.12466	0.000	−1.2713	−0.7816
	4	0.0210	0.12466	0.866	−0.2239	0.2659
2	1	0.7788^a^	0.12466	0.000	0.5339	1.0237
	3	−0.2477^a^	0.12466	0.047	−0.4925	−0.0028
	4	0.7998^a^	0.12466	0.000	0.5549	1.0447
3	1	1.0264^a^	0.12466	0.000	0.7816	1.2713
	2	0.2477^a^	0.12466	0.047	0.0028	0.4925
	4	1.0475^a^	0.12466	0.000	0.8026	1.2923
4	1	−0.0210	0.12466	0.866	−0.2659	0.2239
	2	−0.7998^a^	0.12466	0.000	−1.0447	−0.5549
	3	−1.0475^a^	0.12466	0.000	−1.2923	−0.8026

a P<0.05 indicates a statistically significant difference. 1, CBV ratio between the lesion area and healthy area −1; 2, CBF ratio between the lesion area and healthy area −1; 3, MTT ratio between the lesion area and healthy area −1; 4, TP ratio between the lesion area and healthy area −1. CBV, cerebral blood volume; CBF, cerebral blood flow; MTT, mean transit time; TP, time-to-peak.

**Table III.** Parameter ratios in the ischemic penumbra and the corresponding healthy side area −1.

Ratio	N	Minimum	Maximum	Mean	SD
1	135	0.42	4.07	1.1446	0.49058
2	135	0.38	8.33	1.9233	1.25488
3	135	0.25	9.61	2.1710	1.52981
4	135	0.86	2.75	1.1236	0.19954

1, CBV ratio between the lesion area and healthy area −1; 2, CBF ratio between the lesion area and healthy area −1; 3, MTT ratio between the lesion area and healthy area −1; 4, TP ratio between the lesion area and healthy area −1. CBV, cerebral blood volume; CBF, cerebral blood flow; MTT, mean transit time; TP, time-to-peak; SD, standard deviation.

**Table IV.** Wilcoxon signed ranks test of parameter values in the ischemic penumbra and the corresponding healthy side area.

Parameter	N	Mean rank	Sum of ranks	P-value
CBV2-CBV1				
Negative ranks	7^a^	8.29	58.0	<0.001
Positive ranks	24^b^	18.25	438.0	
Ties	0^c^			
Total	31			
CBF2-CBF1				
Negative ranks	0^d^	0.00	0	
Positive ranks	31^e^	16.00	496.0	
Ties	0^f^			
Total	31			
MTT2-MTT1				
Negative ranks	29^g^	16.57	480.5	
Positive ranks	2^h^	7.75	15.5	
Ties	0^i^			
Total	31			
TP2-TP1				
Negative ranks	28^j^	16.43	460.0	
Positive ranks	3^k^	12.00	36.0	
Ties	0^l^			
Total	31			

CBV1, CBF1, MTT1 and TP1: ischemic penumbra perfusion parameters; CBV2, CBF2, MTT2 and TP2: healthy side area parameters.

a CBV2 < CBV1;

b CBV2 > CBV1;

c CBV2 = CBV1;

d CBF2 < CBF1;

e CBF2 > CBF1;

f CBF2 = CBF1;

g MTT2 < MTT1;

h MTT2 > MTT1;

i MTT2 = MTT1;

j TP2 < TP1;

k TP2 > TP1;

l TP2 = TP1. CBV, cerebral blood volume; CBF, cerebral blood flow; MTT, mean transit time; TP, time-to-peak.

**Table V.** Ratio of parameters in the ischemic penumbra area and the corresponding healthy side area.

Ratio	N	Mean	SD
1	31	0.2578	0.44001
2	31	0.2996	0.42633
3	31	0.6376	0.25439
4	31	3.9747	8.99462

1, CBV ratio between the lesion area and healthy area −1; 2, CBF ratio between the lesion area and healthy area −1; 3, MTT ratio between the lesion area and healthy area −1; 4, TP ratio between the lesion area and healthy area −1. CBV, cerebral blood volume; CBF, cerebral blood flow; MTT, mean transit time; TP, time-to-peak; SD, standard deviation.
